# Albumin-related nutritional indices and all-cause and cause-specific mortality in older adults with severe dysphagia receiving artificial feeding

**DOI:** 10.3389/fnut.2025.1756087

**Published:** 2026-01-22

**Authors:** Jia Wang, Wenxiu Pan

**Affiliations:** 1 Department of Neurology, Xuanhan County People's Hospital, Dazhou, Sichuan, China; 2 Department of Rehabilitation Medicine, Ruijin Hospital, School of Medicine, Shanghai Jiao Tong University, Shanghai, China; 3 Department of Rehabilitation Medicine, Shanghai Ruijin Rehabilitation Hospital, Shanghai, China

**Keywords:** CALLY, CAR, CONUT, dysphagia, LA, mortality, PNI, sepsis

## Abstract

**Background:**

Older adults with severe dysphagia who rely on long-term enteral nutrition face high mortality risk, yet the predictive value of albumin-related nutritional markers—including the C-reactive protein–albumin–lymphocyte (CALLY) score, prognostic nutritional index (PNI), controlling nutritional status (CONUT), C-reactive protein/albumin ratio (CAR), and lymphocyte–albumin (LA)—for all-cause and infection-specific death remains unclear. In addition, direct head-to-head comparisons of these indices' prognostic performance are limited.

**Objective:**

To assess whether albumin-related indices—CALLY, PNI, CONUT, CAR, and LA—are independently associated with overall and infection-specific (sepsis or pneumonia) mortality in older adults with severe dysphagia receiving long-term artificial nutrition.

**Methods:**

In this cohort, we analyzed 236 hospitalized older Japanese patients with severe dysphagia receiving long-term artificial feeding. Associations of CALLY, PNI, CONUT, CAR, and LA with all-cause and infection-related (sepsis or pneumonia) mortality were evaluated using multivariable Cox proportional hazards models, restricted cubic spline analyses and Kaplan–Meier survival curves. Discriminative and incremental predictive performance were assessed by receiver operating characteristic (ROC) curves, net reclassification improvement (NRI) and integrated discrimination improvement (IDI). A mediation analysis tested whether hemoglobin concentration explained the observed associations, and subgroup, sensitivity and competing-risk analyses were performed to verify result robustness.

**Results:**

Higher CALLY, PNI, and LA were independently associated with lower risks of overall and infection-related (sepsis or pneumonia) mortality, whereas higher CONUT scores predicted increased risk. CAR was related to all-cause mortality but showed no significant link with infection-specific death. Restricted cubic spline analyses revealed non-linear dose–response relationships for LA with all-cause mortality and for CONUT with sepsis/pneumonia mortality, while most other associations were approximately linear. The association between LA and sepsis/pneumonia mortality remained significant after competing-risk adjustment. ROC analyses indicated that PNI, CONUT and LA provided incremental discriminatory value compared with albumin alone. Mediation analysis suggested that hemoglobin concentration partially mediated the relationships of CALLY, CAR, and LA with all-cause mortality.

**Conclusions:**

PNI, CONUT, and LA provide incremental prognostic value beyond serum albumin for predicting all-cause mortality. In Japanese elderly patients with severe dysphagia receiving long-term enteral nutrition, LA appears particularly sensitive for identifying infection-related deaths, including sepsis and pneumonia.

## 1 Introduction

Global population aging is progressing rapidly, with the number of very old people rising substantially and those aged over 80 years projected to roughly double in the next three decades (1). Dysphagia is common in older adults (2); its prevalence grows with advancing age and often leads to markedly reduced oral intake. Around 30%−50% of individuals with swallowing dysfunction develop malnutrition, which is associated with impaired immune competence and an increased risk of infection (3, 4). For patients who cannot take nutrition orally, long-term artificial nutrition [for example, gastrostomy or total parenteral nutrition (TPN)] is the primary life-sustaining measure. However, elderly patients on prolonged artificial feeding frequently exhibit hypoalbuminaemia, chronic inflammatory states and immune suppression, predisposing them to infectious complications such as sepsis and pneumonia and contributing to substantially higher overall mortality (5, 6).

Although serum albumin conveys information on both nutritional reserves and systemic inflammation, its value as a lone prognostic indicator is limited (7–9). To better capture the interplay among nutrition, inflammation and immune function, several composite indices have been developed—including C-reactive protein–albumin–lymphocyte (CALLY), prognostic nutritional index (PNI), controlling nutritional status (CONUT), C-reactive protein/albumin ratio (CAR), and lymphocyte–albumin (LA)—which combine albumin with measures such as lymphocyte count, C-reactive protein or cholesterol, and have been linked to mortality risk in various clinical populations (10–14). For example, CALLY has been associated with survival outcomes in older patients with dysphagia (11); PNI has been used to predict post-stroke infections and death (15, 16); CONUT (albumin, cholesterol and lymphocyte count) independently forecasts poor prognosis after acute ischemic stroke (16, 17); and LA has been correlated with overall and disease-free survival in rectal cancer (18).

Nevertheless, the extent to which these composite scores predict overall and infection-specific mortality (notably sepsis and pneumonia) in the high-risk group of older adults with dysphagia dependent on prolonged artificial nutrition is not well-established. Direct, comparative evaluations of CALLY, PNI, CONUT, CAR, and LA in this clinical setting are therefore necessary. In this study, we assessed and contrasted the prognostic performance of these five albumin-related indices for all-cause and infection-related death in a cohort of Japanese elderly patients with dysphagia receiving long-term artificial nutritional support, with the aim of enhancing risk stratification and informing prognostic management in this vulnerable population.

## 2 Materials and methods

### 2.1 Study design and data sources

**Figure 1:**
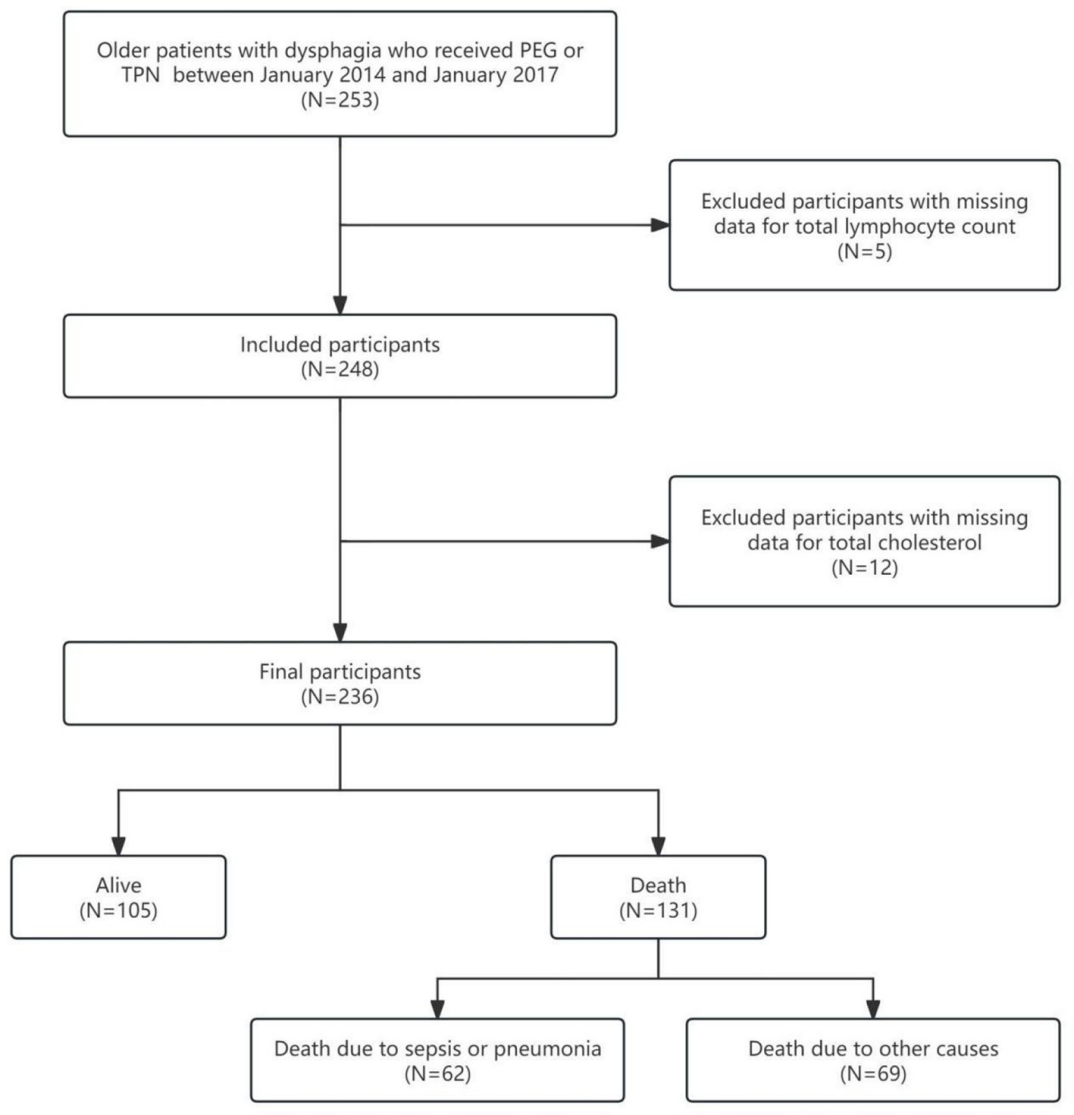
Flowchart of of the selection procedure.

We performed a retrospective cohort study including 236 hospitalized older adults at a single center in Japan between January 2014 and January 2017. Severe dysphagia necessitating artificial nutritional therapy was established by a multidisciplinary team (physicians, nurses and speech-language therapists) and confirmed by videofluoroscopic swallow study. Nutritional support consisted of percutaneous endoscopic gastrostomy (PEG) or total parenteral nutrition (TPN) delivered via an implanted port, a non-tunneled central venous catheter, or a peripherally inserted central catheter. The selection and exclusion process is outlined in Figure 1; patients were excluded if total lymphocyte count or total cholesterol data were unavailable. The protocol was approved by the Ethics Committee of Miyanomori Memorial Hospital, and informed consent was waived because all data were anonymised. Source data were obtained from the Dryad repository; the original data collection had ethical approval and secondary use complied with open-data requirements.

### 2.2 Definition of CALLY, PNI, CONUT, CAR, and LA

Fasting venous blood was obtained within 7 days before the start of artificial nutritional support. Laboratory measurements included hemoglobin (Hb), serum albumin (Alb), C-reactive protein (CRP), total cholesterol (TC) and total lymphocyte count (TLC). Patients were stratified into three tertiles (T1–T3), with T1 used as the reference category. The composite nutritional indices were calculated as follows:

*CALLY* = [*Albumin* (*g*/*dl*) × *Total lymphocyte count* (*cells*/*mm*^3^)]/[*CRP* (*mg*/*dl*) × 10]

*PNI* = 10 × *Albumin* (*g*/*dl*) + 0.005 × *Total lymphocyte count* (*cells*/*mm*^3^)

CONUT score = sum of points for albumin, total lymphocytes and total cholesterol, assigned as:

*Albumin*: ≥ 3.5 *g*/*dl* = 0; 3.03.4 *g*/*dl* = 2; 2.52.9 *g*/*dl* = 4; < 2.5 *g*/*dl* = 6

*Total lymphocyte count*: ≥ 1, 600 *cells*/*mm*^3^ = 0; 1, 2001, 599 = 1; 8001199 = 2; < 800 = 3

*Total cholesterol*: ≥ 180 *mg*/*dl* = 0; 140179 = 1; 100139 = 2; < 100 = 3

*CAR* = *Albumin* (*g*/*dl*)/*CRP* (*mg*/*dl*)

*LA* = *Albumin* (*g*/*L*) × *Total lymphocyte count* (10^9^/*L*)

### 2.3 Outcomes

The primary endpoint was all-cause mortality occurring during the follow-up period after initiation of long-term artificial nutritional support. The secondary endpoint was infection-related death (severe pneumonia or sepsis), with causes of death adjudicated by treating physicians based on clinical evaluation.

### 2.4 Variables

The route of artificial nutrition was chosen through discussion between the treating physician, the patient and/or their family, and the specific feeding regimen was prescribed at the clinician's discretion. Clinical and laboratory data were retrospectively extracted from medical records and comprised demographic information (age, sex), comorbid conditions (e.g., cerebrovascular disease, advanced dementia, aspiration pneumonia, ischemic heart disease) and baseline blood tests (serum albumin, total lymphocyte count, hemoglobin, total cholesterol, and C-reactive protein). For analyses, laboratory results obtained within 7 days before initiation of percutaneous endoscopic gastrostomy (PEG) or total parenteral nutrition (TPN) were used. Daily caloric intake on post-operative day 7 was recorded for both PEG and TPN recipients, and frailty was assessed using the Clinical Frailty Scale at the time of gastrostomy or at the start of parenteral nutrition.

### 2.5 Data processing

The extent of missing data is summarized in Supplementary Table S1. Prior to the main analyses, all variables were evaluated for multicollinearity, with a generalized variance inflation factor ≥2 taken to indicate meaningful multicollinearity (see Supplementary Table S2). Because missingness affected under 5% of observations, a complete-case approach was used for the primary analyses, excluding records with any missing values.

### 2.6 Statistical analysis

Continuous variables that approximated a normal distribution are reported as mean ± standard deviation, while those with skewed distributions are presented as median (interquartile range). Categorical variables are shown as counts with corresponding percentages. The CALLY variable exhibited left skew and was therefore log-transformed (natural log) for subsequent analyses (19). Each nutritional index (CALLY, PNI, CONUT, CAR, and LA) was used to divide participants into three tertiles (T1–T3). Survival across tertiles for both all-cause and infection-related deaths was compared using Kaplan–Meier curves. We fitted multivariable Cox proportional-hazards models to estimate hazard ratios (HRs) with 95% confidence intervals (CIs) for the associations between each index and mortality outcomes. Two models were prespecified: Model 1 (unadjusted) and Model 2, the primary model, which adjusted for age, sex, comorbidities (cerebrovascular disease, dementia, ischemic heart disease, aspiration pneumonia), nutritional status (hemoglobin), type of nutritional support (PEG, TPN, or return to oral intake) and clinical frailty score. Dose–response relationships were examined using restricted cubic spline analyses with four knots placed at the 5th, 35th, 65th, and 95th percentiles, accounting for the covariates included in Model 2.

The discriminatory performance of the five indices for incident events during follow-up was evaluated by receiver operating characteristic (ROC) curve analysis. Incremental predictive value beyond albumin was assessed using net reclassification improvement (NRI) and integrated discrimination improvement (IDI). Sensitivity analyses included: (1) repeating the main analyses on the original dataset including cases with missing data; (2) excluding patients who died in hospital within 30 days to reduce reverse-causation bias; (3) stratified analyses to explore effect modification by age (≥90 vs. < 90 years), sex, and nutritional support modality (PEG vs. TPN), with likelihood-ratio tests for subgroup interactions; and (4) Fine–Gray subdistribution hazard models to handle competing risks, treating deaths not due to sepsis or pneumonia as competing events (Supplementary Tables S3–S5). All analyses were performed in R v4.2.2 and Free Statistics v2.2. Two-sided *p* < 0.05 was considered statistically significant.

## 3 Results

### 3.1 Baseline demographic and clinical characteristics of older adults with dysphagia

**Figure 2:**
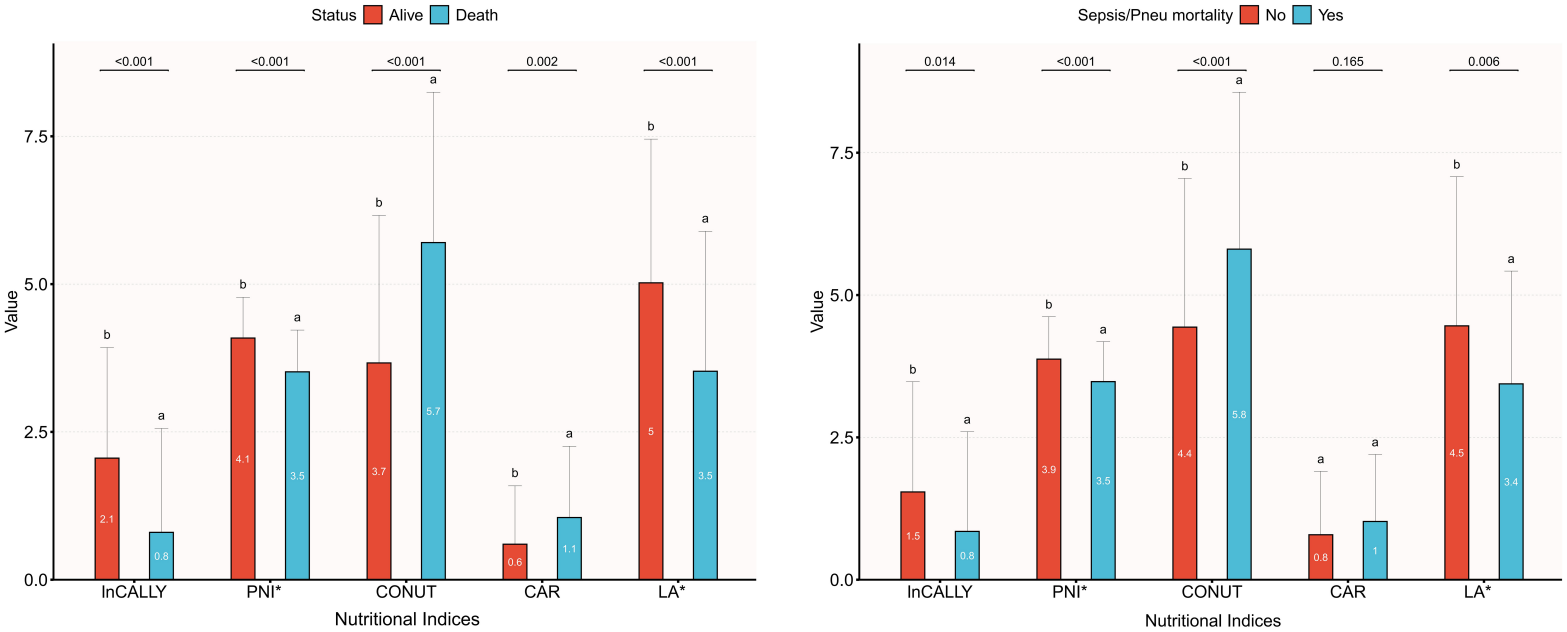
Mean values of five clinical indices by survival status and infection-related cause of death (sepsis or pneumonia). Bar chart presenting group means for InCALLY, PNI^*^, CONUT, CAR, and LA^*^ stratified by survival status (Alive vs. Death) in **(a)** and by deaths attributed to sepsis or pneumonia in **(b)**. Mean values for Alive vs. Death are: InCALLY 2.1 vs. 0.8; PNI^*^ 4.1 vs. 3.5; CONUT 3.7 vs. 5.7; CAR 0.6 vs. 1.1; LA^*^ 5.0 vs. 3.5. Two-sided *p* values for the Alive vs. Death comparisons (left to right) are: < 0.001, < 0.001, < 0.001, 0.002, < 0.001. Different lowercase letters shown above bars indicate significant pairwise differences (different letters mean *p* < 0.05). ^*^PNI and LA are expressed per standard deviation.

Table 1 reports baseline characteristics according to survival status and whether death was due to sepsis or pneumonia. After excluding five patients without lymphocyte counts and 12 lacking cholesterol measurements, the final sample comprised 236 individuals with dysphagia (mean age 82.8 years, SD 9.3; 40.3% male), censored participants had a median follow-up of 314 days. There were 131 deaths overall, of which 62 were ascribed to severe pneumonia or sepsis. Characteristics of excluded vs. included participants are shown in Supplementary Table S1. Figure 2 compares the distributions of five indices (InCALLY, PNI, CONUT, CAR, and LA) between survivors and non-survivors, with infection-related deaths separated by sepsis or pneumonia. InCALLY, PNI, CONUT and LA differed significantly between groups (exact means and *p*-values are presented in the figure); non-survivors had substantially higher CONUT scores, while survivors exhibited higher InCALLY and PNI. CAR also showed a significant difference (*p* = 0.002). Lowercase letters above the bars denote pairwise differences (different letters indicate *p* < 0.05). Group comparisons were performed using Student's *t*-test or the Mann–Whitney *U* test, as appropriate; all tests were two-sided and a *p* value < 0.05 was considered significant. Both all-cause and infection-related mortality were significantly associated with male sex, cerebrovascular disease, advanced dementia, greater frailty, aspiration pneumonia, a lower likelihood of resuming oral feeding, lower hemoglobin, decreased CALLY, PNI and LA, and elevated CONUT and CAR (all *p* < 0.05). By contrast, infection-related mortality did not differ significantly by age, presence of ischemic heart disease, or nutritional support modality (*p* > 0.05).

**Table 1:** Baseline characteristics of patients.

**Variables**	**Total (*n* = 236)**	**All-cause mortality**			**Sepsis or pneumonia mortality**		
		**No (*****n*** = **105)**	**Yes (*****n*** = **131)**	***p*** **-Value**	**No (*****n*** = **174)**	**Yes (*****n*** = **62)**	***p*** **-Value**
Age, Mean ± SD	82.8 ± 9.3	79.8 ± 10.2	85.3 ± 7.7	<0.001	82.4 ± 9.8	84.1 ± 7.9	0.214
**Sex**, ***n*** **(%)**					
Male	95 (40.3)	27 (25.7)	68 (51.9)	<0.001	56 (32.2)	39 (62.9)	<0.001
Female	141 (59.7)	78 (74.3)	63 (48.1)		118 (67.8)	23 (37.1)	
CVD, *n* (%)	126 (53.4)	67 (63.8)	59 (45)	0.004	102 (58.6)	24 (38.7)	0.007
Dementia, *n* (%)	97 (41.1)	31 (29.5)	66 (50.4)	0.001	64 (36.8)	33 (53.2)	0.024
**CFS**, ***n*** **(%)**					
CFS < 8	37 (15.7)	30 (28.6)	7 (5.3)	<0.001	36 (20.7)	1 (1.6)	<0.001
CFS≥8	199 (84.3)	75 (71.4)	124 (94.7)		138 (79.3)	61 (98.4)	
Asp, *n* (%)	90 (38.1)	30 (28.6)	60 (45.8)	0.007	53 (30.5)	37 (59.7)	<0.001
IHD, *n* (%)	42 (17.8)	11 (10.5)	31 (23.7)	0.008	27 (15.5)	15 (24.2)	0.125
PEG, *n* (%)	169 (71.6)	96 (91.4)	73 (55.7)	<0.001	128 (73.6)	41 (66.1)	0.265
TPN, *n* (%)	69 (29.2)	32 (30.5)	37 (28.2)	0.708	50 (28.7)	19 (30.6)	0.777
Oral, *n* (%)	14 ( 5.9)	14 (13.3)	0 (0)	<0.001	14 (8)	0 (0)	0.024
Hemoglobin (g/dl), Mean ± SD	11.0 ± 2.0	11.8 ± 1.8	10.3 ± 2.0	<0.001	11.2 ± 2.0	10.3 ± 2.0	0.002
**Nutritional indices**					
CALLY, Median (IQR)	3.8 (0.8, 15.9)	10.0 (1.9, 32.2)	1.9 (0.6, 6.9)	<0.001	4.7 (1.0, 18.9)	1.9 (0.6, 7.7)	0.020
PNI, Mean ± SD	37.7 ± 7.5	40.9 ± 6.9	35.2 ± 7.1	<0.001	38.7 ± 7.5	34.8 ± 7.0	<0.001
CONUT, Mean ± SD	4.8 ± 2.7	3.7 ± 2.5	5.7 ± 2.5	<0.001	4.4 ± 2.6	5.8 ± 2.8	<0.001
CAR, Median (IQR)	0.3 (0.1, 1.2)	0.2 (0.1, 0.6)	0.6 (0.2, 1.5)	<0.001	0.3 (0.1, 1.0)	0.6 (0.2, 1.5)	0.045
LA, Mean ± SD	41.9 ± 25.0	50.2 ± 24.3	35.3 ± 23.7	<0.001	44.6 ± 26.2	34.4 ± 19.8	0.006

SD, standard deviation; IQR, interquartile range; CVD, cerebrovascular diseases; Dementia, severe dementia; CFS, Clinical Frailty Scale; Asp, aspiration pneumonia; IHD, ischemic heart disease; PEG, percutaneous endoscopic gastrostomy; TPN, total parenteral nutrition; Oral, oral intake recovery; CALLY,C-reactive protein-albumin-lymphocyte index; PNI, prognostic nutritional index; CONUT, controlling nutritional status; CAR, C-reactive protein/albumin ratio; LA, lymphocyte-to-albumin.

### 3.2 Associations of five indices with all-cause and sepsis/pneumonia-related mortality

**Figure 3:**
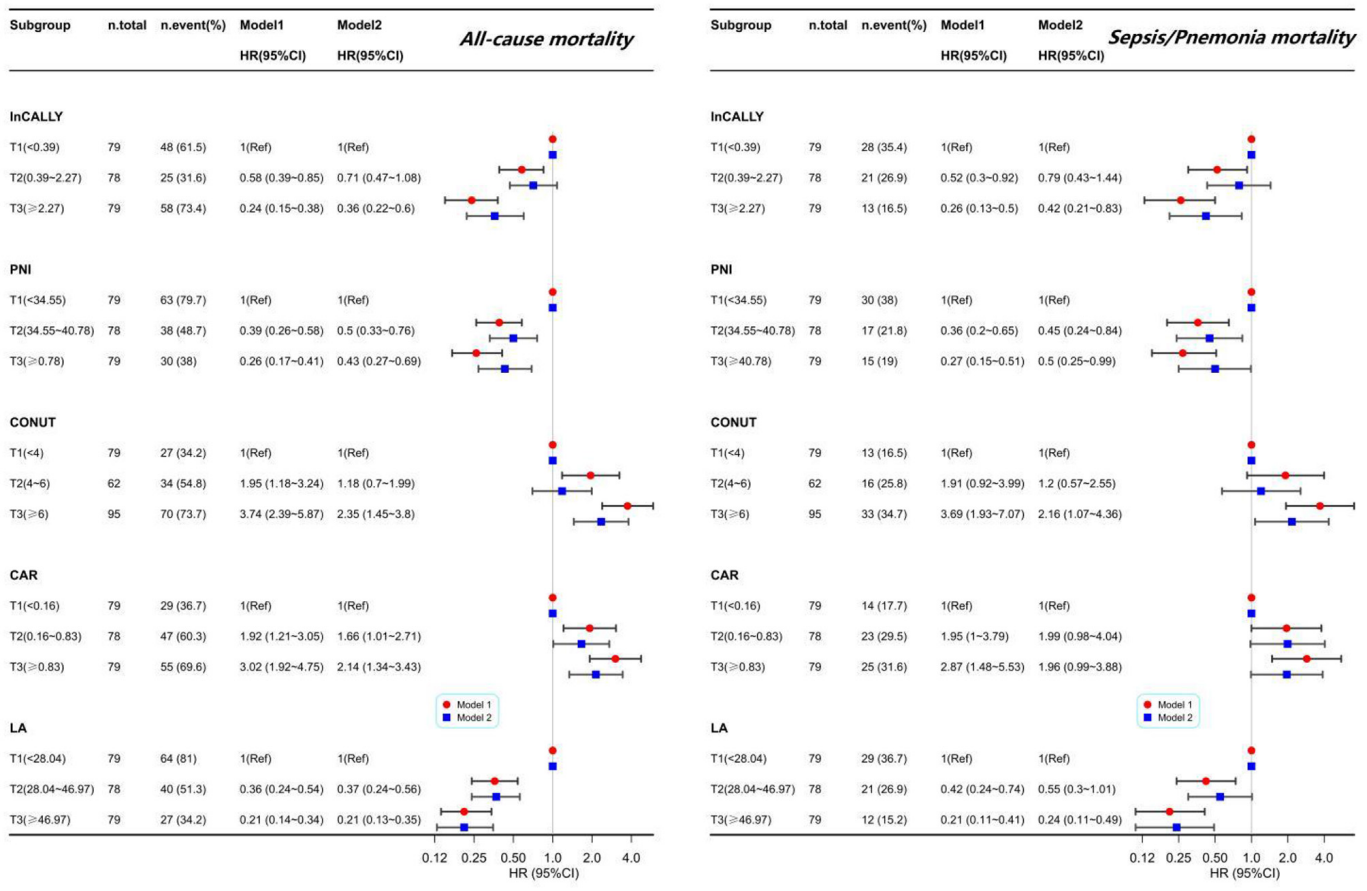
Hazard ratios for tertiles of nutritional indices from multivariable Cox models: all-cause and sepsis/pneumonia mortality. Forest plot showing hazard ratios (HRs) with 95% confidence intervals (CIs) derived from multivariable Cox proportional-hazards analyses for tertiles (T1–T3) of five indices (InCALLY, PNI, CONUT, CAR, LA). The upper panel displays results for all-cause mortality, and the lower panel for deaths due to sepsis or pneumonia. For each subgroup, the left-hand table gives the total number of patients and the number (%) of events; T1 is used as the reference category (HR = 1.00). Model 1 presents unadjusted estimates, while Model 2 is fully adjusted for sex, age, cerebrovascular disease, dementia, ischemic heart disease, aspiration pneumonia, nutritional support modality (PEG, TPN, or return to oral intake) and the clinical frailty score. HRs (with 95% CIs) are plotted on a log scale; intervals that cross 1.00 indicate no statistically significant association at the 0.05 threshold.

The adjusted multivariable forest plot in Figure 3 illustrates the relationships between the five indices and mortality outcomes; full effect estimates appear in Supplementary Tables S3, S4. After adjusting for covariates, higher CALLY, PNI and LA were independently associated with lower hazards of both all-cause death and death from sepsis or pneumonia, whereas higher CONUT predicted increased risk. CAR showed a positive association only with overall mortality (*p* for trend < 0.05) and was not significantly related to sepsis/pneumonia deaths. Of the indices with inverse associations, LA displayed the strongest protective effect (all-cause HR 0.21, 95% CI 0.13–0.35; sepsis/pneumonia HR 0.24, 95% CI 0.11–0.49), followed by CALLY (all-cause HR 0.36, 95% CI 0.22–0.60; sepsis/pneumonia HR 0.42, 95% CI 0.21–0.83). For indices associated with increased mortality, the point estimate for CONUT was greater than that for CAR.

**Figure 4:**
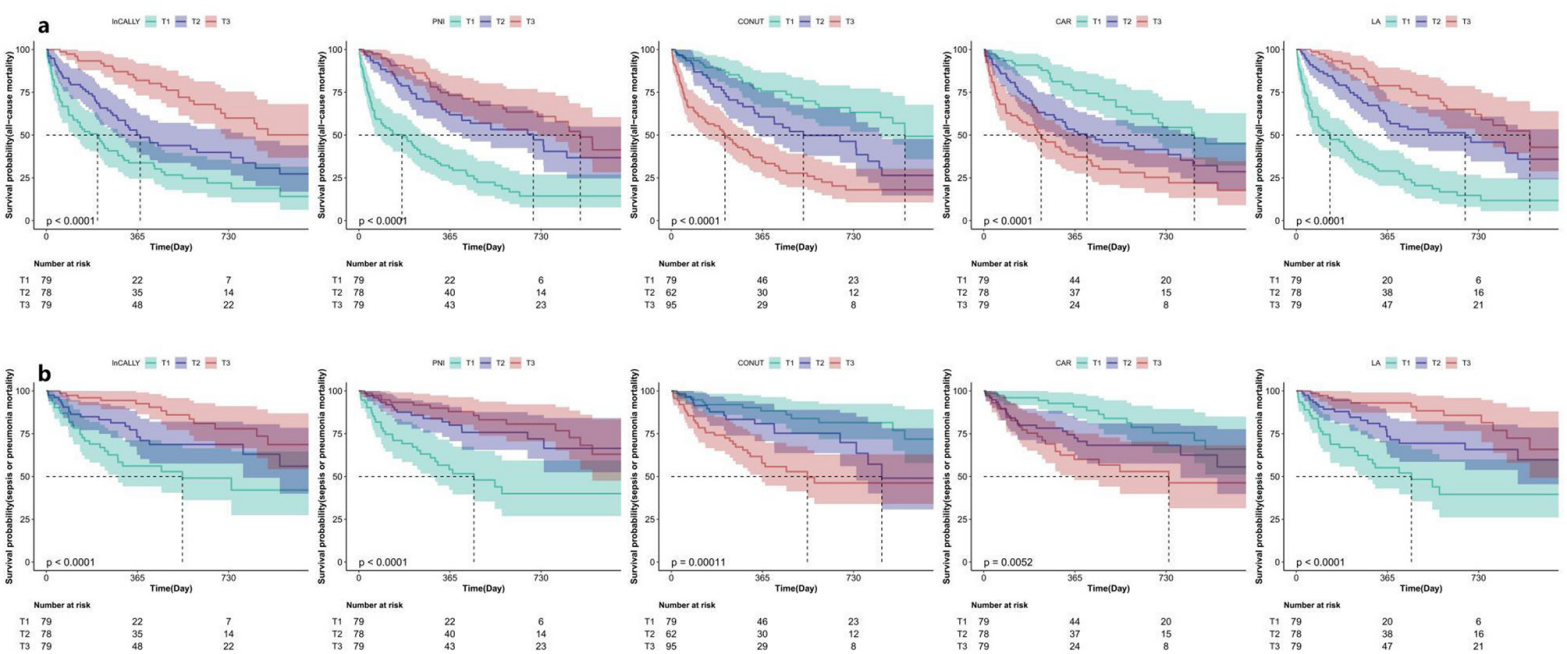
Kaplan–Meier survival curves for all-cause and sepsis/pneumonia mortality by tertiles of five nutritional indices. Kaplan–Meier curves depict survival differences across tertiles (T1 = lowest, T3 = highest) of baseline InCALLY, PNI, CONUT, CAR and LA. Panel **a** presents all-cause mortality and panel b shows deaths due to sepsis or pneumonia. The horizontal axis indicates follow-up time in days (0, 365, 730) and the vertical axis shows the estimated probability of survival. Group comparisons were performed with the log-rank test and the corresponding *p*-values are annotated on the plots. For all-cause mortality (panel **a**) each index reached strong statistical significance (*p* < 0.0001). In panel **b**, *p*-values were < 0.0001 for InCALLY, PNI and LA, 0.00011 for CONUT, and 0.0052 for CAR. Counts of patients at risk by tertile at baseline and at 365 and 730 days are listed beneath each panel. Details on how T1–T3 were defined and the operational definitions of the indices are provided in the **Methods** section.

**Figure 5:**
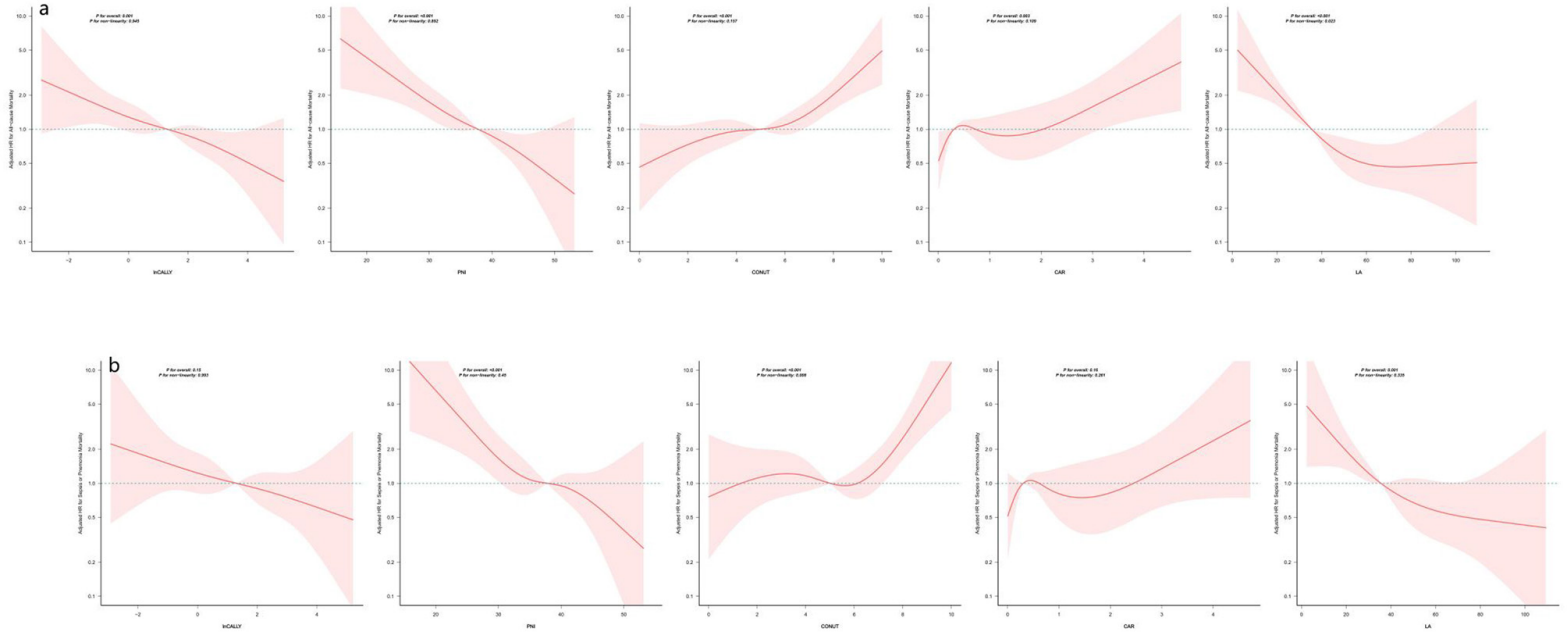
Restricted cubic spline analyses of five indices in relation to all-cause and sepsis/pneumonia mortality. The panels display smoothed, multivariable-adjusted hazard ratios (solid lines) with 95% confidence bands (shaded areas) for all-cause mortality (panel **a**) and for sepsis/pneumonia mortality (panel **b**) across the continuous range of each nutritional/inflammatory marker. Estimates come from Cox models adjusted for sex, age, cerebrovascular disease, dementia, ischemic heart disease, aspiration pneumonia, nutritional support modality (PEG, TPN, or oral intake) and clinical frailty score. The dashed horizontal line marks HR = 1. Reported *p*-values include tests for the overall association and for departure from linearity; the former assesses whether the marker relates to the outcome, while the latter evaluates whether that relationship is non-linear. In this analysis, each marker showed a statistically significant overall association (*p* for overall < 0.001), and non-linearity tests were >0.05, indicating the effect can be well-approximated by a linear relation. HRs above 1 indicate higher marker levels are associated with increased mortality risk, whereas HRs below 1 indicate lower risk. Curves are plotted only across the ranges commonly observed in the sample; estimates beyond those ranges may be unstable. Abbreviations are defined in the Methods.

Kaplan-Meier plots indicate a tertile-dependent reduction in mortality for CALLY, PNI, and LA, in contrast to increasing mortality across tertiles for CONUT and CAR; all differences across groups were significant by the log-rank test (*p* < 0.05) (Figure 4). Figure 5 shows restricted cubic spline curves modeling the associations between CALLY, PNI, CONUT, CAR, and LA and the mortality outcomes. After adjustment for the covariates in Model 2, spline testing indicated that CALLY, PNI, CONUT, and CAR had associations with all-cause mortality that were essentially linear (overall *P* < 0.05; *P* for non-linearity > 0.05). For sepsis/pneumonia mortality, PNI and LA likewise followed linear patterns (overall *P* < 0.05; *P* for non-linearity > 0.05). In contrast, LA displayed a significant non-linear relationship with all-cause mortality (overall *P* < 0.05; *P* for non-linearity < 0.05), and CONUT showed a non-linear association with sepsis/pneumonia mortality (overall *P* < 0.05; *P* for non-linearity < 0.05).

### 3.3 Mediation analyses of CALLY, PNI, CONUT, CAR, and LA in relation to all-cause and sepsis/pneumonia mortality

Mediation analyses indicated that hemoglobin partially mediated the associations of CALLY, CAR, and LA with all-cause mortality, accounting for 17.75% of the total effect for CALLY, 30.93% for CAR and 9.74% for LA (Supplementary Table S5). By contrast, there was no evidence that hemoglobin mediated the relationships with sepsis/pneumonia-specific mortality.

### 3.4 Receiver operating characteristic (ROC) curve analysis was performed to assess the discriminatory performance of CALLY, PNI, CONUT, CAR, and LA for all-cause and sepsis/pneumonia mortality

ROC analyses indicated that PNI provided the best discrimination for all-cause mortality (AUC 0.7211, 95% CI 0.6561–0.7862), with CONUT (AUC 0.7117, 95% CI 0.6468–0.7766) and LA (AUC 0.7082, 95% CI 0.6421–0.7742) close behind (Supplementary Tables S6, S7; Supplementary Figures S1, S2). For deaths due to sepsis or pneumonia, PNI again showed the highest AUC (0.6433, 95% CI 0.5651–0.7216), followed by CONUT (0.6398, 95% CI 0.5578–0.7219) and LA (0.6202, 95% CI 0.5407–0.6997). Because these indices combine serum albumin with additional laboratory measures, we evaluated their incremental predictive value over albumin alone using net reclassification improvement (NRI) and integrated discrimination improvement (IDI). Except for CAR, all indices produced a significant NRI for all-cause mortality (*p* < 0.05); additionally, PNI, CONUT and LA achieved significant gains in IDI (*p* < 0.05). Regarding sepsis/pneumonia mortality, only PNI (IDI 0.031, 95% CI 0.001–0.084) and CONUT (IDI 0.033, 95% CI 0.002–0.111) showed statistically significant IDI increases (*p* < 0.05).

### 3.5 Sensitive analysis

We assessed the stability of our main results through several sensitivity analyses. First, after applying competing-risk methods, only LA preserved comparable associations with both overall and sepsis/pneumonia mortality (Table 2). Second, findings were similar when the analyses were limited to complete-case data and when the original dataset that retained missing observations was re-analyzed (Supplementary Table S8). Third, excluding patients who died within 30 days of admission did not materially change the associations between the five indices and either all-cause or infection-related mortality (Supplementary Table S9). Fourth, stratified analyses by age, sex and type of nutritional support were performed and likelihood-ratio tests were used to evaluate effect modification; an interaction with TPN was detected for CALLY and CAR, while no other subgroup showed significant interactions (Supplementary Table S10).

**Table 2:** Associations of nutritional/inflammatory indices with all-cause mortality by Fine–Gray competing risks regression.

**Variable**	***n*.total**	***n*.event (%)**	**Fine-Gray**			
			**Model1**		**Model2**	
			**HR (95%CI)**	***p*** **-Value**	**HR (95%CI)**	***p*** **-Value**
***lnCALLY***						
lnCALLY	236.0	62 (26.3)	0.84 (0.74–0.95)	0.007	0.91 (0.79–1.04)	0.155
T1 (< 0.39)	79.0	28 (35.4)	1 (Ref)		1 (Ref)	
T2 (0.39–2.27)	78.0	21 (26.9)	0.66 (0.37–1.17)	0.158	0.91 (0.51–1.62)	0.747
T3 (≥2.27)	79.0	13 (16.5)	0.39 (0.21–0.74)	0.004	0.58 (0.3–1.12)	0.106
*p* for trend				0.003		0.116
***PNI***						
PNI	236.0	62 (26.3)	0.62 (0.49–0.79)	<0.001	0.68 (0.51–0.89)	0.006
T1 (< 34.55)	79.0	30 (38)	1 (Ref)		1 (Ref)	
T2 (34.55–40.78)	78.0	17 (21.8)	0.52 (0.29–0.94)	0.031	0.56 (0.3–1.04)	0.065
T3 (≥40.78)	79.0	15 (19)	0.44 (0.24–0.8)	0.007	0.67 (0.35–1.26)	0.215
*p* for trend				0.007		0.166
***CONUT***						
CONUT	236.0	62 (26.3)	1.2 (1.08–1.33)	0.001	1.16 (1.03–1.3)	0.014
T1 (< 4)	79.0	13 (16.5)	1 (Ref)		1 (Ref)	
T2 (4–6)	62.0	16 (25.8)	1.66 (0.82–3.37)	0.162	1.31 (0.66–2.61)	0.442
T3 (≥6)	95.0	33 (34.7)	2.42 (1.29–4.56)	0.006	1.63 (0.83–3.2)	0.152
*p* for trend				0.005		0.148
***CAR***						
CAR	236.0	62 (26.3)	1.16 (0.97–1.39)	0.11	1.02 (0.82–1.27)	0.86
T1 (< 0.16)	79.0	14 (17.7)	1 (Ref)		1 (Ref)	
T2 (0.16–0.83)	78.0	23 (29.5)	1.75 (0.91–3.34)	0.092	1.77 (0.89–3.52)	0.104
T3 (≥0.83)	79.0	25 (31.6)	2 (1.06–3.77)	0.031	1.44 (0.75–2.74)	0.273
*p* for trend				0.029		0.334
***LA***						
LA^*^	236.0	62 (26.3)	0.63 (0.45–0.88)	0.006	0.68 (0.48–0.96)	0.027
T1 (< 28.04)	79.0	29 (36.7)	1 (Ref)		1 (Ref)	
T2 (28.04–46.97)	78.0	21 (26.9)	0.66 (0.37–1.16)	0.149	0.88 (0.5–1.53)	0.649
T3 (≥46.97)	79.0	12 (15.2)	0.36 (0.18–0.68)	0.002	0.47 (0.24–0.94)	0.034
*p* for trend				0.002		0.036

Model1: Crude Model, Model2: adjusted for sex, age, cerebrovascular diseases, severe dementia, oral intake, clinical frailty scale.

PNI^*^, PNI per SD; LA^*^, LA per SD.

## 4 Discussion

Our main findings are summarized as follows. (1) CAR showed no significant association with infection-related mortality, whereas CALLY, PNI and LA were inversely associated with both all-cause and infection-related mortality (sepsis or pneumonia); by contrast, higher CONUT indicated increased risk. LA demonstrated the largest effect estimate for both overall and infection-related mortality, and this relationship remained robust after adjustment for competing risks. CAR was associated only with all-cause mortality. (2) ROC analyses showed that PNI, CONUT, and LA discriminated both all-cause and infection-related death better than albumin alone. (3) Hemoglobin partially mediated the associations of CALLY, CAR, and LA with all-cause mortality. (4) The inverse associations of CALLY and CAR with all-cause mortality were more evident among patients not receiving TPN, while the associations of PNI, CONUT and LA with all-cause mortality were consistent across age, sex and nutritional-support subgroups.

In this cohort of older adults with severe dysphagia requiring prolonged artificial nutritional support, higher CALLY and PNI were associated with lower mortality and elevated CAR and CONUT with greater risk, in agreement with prior reports. After covariate adjustment, CALLY, PNI, CONUT, and LA were each significantly associated with death from sepsis or pneumonia; LA remained robust when competing risks were considered, indicating a particularly strong prognostic signal. Head-to-head comparisons showed that, apart from CAR, each composite index improved net reclassification over albumin for all-cause mortality, with PNI, CONUT, and LA also improving integrated discrimination. For sepsis- or pneumonia-related mortality, only PNI and CONUT improved IDI compared with albumin. Restricted cubic spline analysis identified predominantly linear dose–response relationships for most indices, with LA exhibiting a non-linear pattern.

Hemoglobin acted as a partial mediator, with indirect effect proportions of 17.75% (CALLY), 30.93% (CAR) and 9.74% (LA). These findings are compatible with an inflammation-driven impairment of iron metabolism and erythropoiesis (e.g., hepcidin-mediated pathways) leading to anemia, which then impairs oxygen delivery and exacerbates organ dysfunction, thereby increasing mortality risk (20–25). Although mediation estimates support this pathway, causal inference is limited by the observational design; interventional studies are needed to establish whether correcting anemia or upstream inflammation reduces mortality in this population (26–28).

Biologically and methodologically, composite indices outperform solitary biomarkers because they integrate information on nutrition, inflammation and immune status, mitigating floor effects and transient variability inherent to single measures such as albumin or CRP. Our analyses using Kaplan–Meier curves, Cox models and restricted cubic splines confirm that these indices independently predict mortality in older patients with dysphagia, consistent with previous literature (11, 13, 18, 29, 30). Pathophysiologically, malnutrition and systemic inflammation plausibly impair host defense, promote oxidative stress and disrupt microbiota, accelerating multisystem decline and increasing vulnerability to severe infections (31).

The LA index shows potential as a simple, readily available marker that provides prognostic information incremental to albumin alone, particularly for infection-related mortality and hemoglobin emerges as a potential modifiable intermediary. However, this is an exploratory finding that requires external validation in independent cohorts and prospective evaluation to establish reproducibility, discrimination and added clinical utility. Subgroup analyses showed that PNI, CONUT and LA predicted outcomes irrespective of PEG or TPN use, whereas CALLY and CAR displayed effect modification by TPN, which may reflect modality-specific infection risks or selection bias and warrants cautious interpretation (32). Findings were otherwise consistent across age and sex strata.

We simultaneously evaluated the diagnostic and prognostic accuracy of several indices and examined how Hb alters the associations of each individual index and their combined measures. Because these scores are calculated from routine, rapidly available laboratory tests (CRP, albumin, lymphocyte count and cholesterol), they impose minimal additional cost and can be readily integrated into clinical workflows. Applied to older patients with dysphagia receiving prolonged nutritional support, they facilitate low-cost risk stratification and offer actionable guidance for nutritional management. Methodological strengths of this study—including direct head-to-head comparisons, mediation analysis to interrogate mechanisms, and the use of competing-risks models and restricted cubic splines—enhance the robustness and interpretability of the findings.

Several limitations warrant mention. First, this was a single-center investigation that enrolled a relatively small sample of elderly Japanese patients receiving prolonged enteral nutrition for dysphagia, which restricts generalisability. Second, all indices were calculated from a single baseline blood draw, so we could not assess how changes over time relate to long-term outcomes. Third, despite sensitivity analyses, residual confounding from unmeasured factors cannot be excluded—for example, disease-specific severity scores, baseline infection status and antibiotic exposure, immunosuppressive therapy, device use, and measures of organ function. Finally, the observational design prevents causal inference; confirmation will require larger, multicentre prospective studies. Future work should compare the LA index with established scores such as the Sequential Organ Failure Assessment (SOFA) and quick SOFA (qSOFA), and evaluate its clinical utility (for instance by decision-curve analysis) in adequately powered prospective cohorts.

## 5 Conclusion

Among older Japanese patients with severe dysphagia dependent on long-term artificial feeding, the indices CALLY, PNI, CONUT, CAR, and LA were each significantly associated with overall mortality and with deaths due to infection. Elevated CONUT and CAR values predicted a higher risk of death, whereas greater CALLY, PNI, and LA scores were associated with lower mortality. LA, notably, showed the highest sensitivity for identifying fatalities attributable to sepsis or pneumonia. Given that LA is easy to calculate and requires no specialized equipment, it could be adopted as a pragmatic screening metric to flag elderly recipients of artificial nutrition at increased risk of infection-related death, thereby facilitating earlier targeted interventions and preventive measures.

## Data Availability

The original contributions presented in the study are included in the article/Supplementary material, further inquiries can be directed to the corresponding author.
