# Supplementary material for: Albumin-related nutritional indices and all-cause and cause-specific mortality in older adults with severe dysphagia receiving artificial feeding
Source: Front Nutr. 2026 Jan 22;12:1756087. doi: 10.3389/fnut.2025.1756087 (PMC12872515; doi:10.3389/fnut.2025.1756087)
Supplement: Supplementary file 1 [file Supplementary_file_1.docx]

**Table S1. Baseline characteristics of the study population: comparison between participants included in the analysis and those excluded.**

| **Variables** | **Total (n = 253)** | **Excluded (n = 17)** | **Included (n = 236)** | ***P*-Value** |
| --- | --- | --- | --- | --- |
| Age, Mean ± SD | 83.1 ± 9.3 | 86.0 ± 9.5 | 82.8 ± 9.3 | 0.18 |
| Sex, n (%) |  |  |  | 0.172 |
| Male | 99 (39.1) | 4 (23.5) | 95 (40.3) |  |
| Female | 154 (60.9) | 13 (76.5) | 141 (59.7) |  |
| CVD, n (%) | 133 (52.6) | 7 (41.2) | 126 (53.4) | 0.33 |
| Dementia, n (%) | 102 (40.3) | 5 (29.4) | 97 (41.1) | 0.343 |
| CFS, n(%) |  |  |  | 0.482 |
| CFS<8 | 38 (15.0) | 1 (5.9) | 37 (15.7) |  |
| CFS=8 | 215 (85.0) | 16 (94.1) | 199 (84.3) |  |
| Asp, n (%) | 94 (37.2) | 4 (23.5) | 90 (38.1) | 0.229 |
| IHD, n (%) | 47 (18.6) | 5 (29.4) | 42 (17.8) | 0.328 |
| Hemoglobin(g/dl), Mean ± SD | 11.0 ± 2.0 | 11.2 ± 1.9 | 11.0 ± 2.0 | 0.696 |
| Oral, n (%) | 15 ( 5.9) | 1 (5.9) | 14 (5.9) | 1 |
| PEG, n (%) | 180 (71.1) | 11 (64.7) | 169 (71.6) | 0.583 |
| TPN, n (%) | 73 (28.9) | 4 (23.5) | 69 (29.2) | 0.784 |
| CALLY, Median (IQR) | 3.8 (0.8, 16.8) | 4.3 (0.0, 19.0) | 3.8 (0.8, 15.9) | 0.693 |
| PNI, Mean ± SD | 37.7 ± 7.5 | 38.4 ± 7.3 | 37.7 ± 7.5 | 0.725 |
| **CONUT, Mean ± SD** | 4.6 ± 2.7 | 2.5 ± 2.1 | 4.8 ± 2.7 | **< 0.001** |
| CAR, Median (IQR) | 0.3 (0.1, 1.1) | 0.3 (0.1, 0.5) | 0.3 (0.1, 1.2) | 0.61 |
| LA, Mean ± SD | 42.5 ± 24.9 | 53.7 ± 18.8 | 41.9 ± 25.0 | 0.111 |
| Status, n (%) |  |  |  | 0.252 |
| Alive | 115 (45.5) | 10 (58.8) | 105 (44.5) |  |
| Death | 138 (54.5) | 7 (41.2) | 131 (55.5) |  |
| Death_pneumonia, n (%) | 44 (17.4) | 1 (5.9) | 43 (18.2) | 0.321 |
| Death_sepsis, n (%) | 20 ( 7.9) | 1 (5.9) | 19 (8.1) | 1 |

Abbreviation: SD, standard deviation; IQR, interquartile range; CVD, cerebrovascular diseases; Dementia, severe dementia; CFS, Clinical Frailty Scale; Asp, aspiration pneumonia; IHD, ischemic heart disease; PEG, percutaneous endoscopic gastrostomy; TPN, total parenteral nutrition; Oral, oral intake recovery; CALLY,C-reactive protein-albumin-lymphocyte index;PNI, prognostic nutritional index;CONUT,controlling nutritional status;CAR,C-reactive protein/albumin ratio;LA,Lymphocyte-to-Albumin.

**Table S2. Multicollinearity assessment**

| **Variables/VIF** | **Crude** | **Age** | **Sex** | **CI** | **Dement** | **Asp** | **IHD** | **Oral** | **PEG** | **TPN** | **CFS** |
| --- | --- | --- | --- | --- | --- | --- | --- | --- | --- | --- | --- |
| ***All-cause mortality*** | | | | | | | | | | | |
| CALLY | 1.047 | 1.31 | 1.276 | 1.659 | 1.795 | 1.477 | 1.092 | 1.0 | 1.393 | 1.136 | 1.044 |
| PNI | 1.04 | 1.26 | 1.259 | 1.642 | 1.733 | 1.504 | 1.105 | 1.0 | 1.292 | 1.209 | 1.075 |
| CONUT | 1.039 | 1.236 | 1.277 | 1.614 | 1.69 | 1.481 | 1.089 | 1.0 | 1.334 | 1.191 | 1.089 |
| CAR | 1.058 | 1.356 | 1.295 | 1.71 | 1.816 | 1.496 | 1.103 | 1.0 | 1.41 | 1.144 | 1.053 |
| LA | 1.097 | 1.259 | 1.222 | 1.676 | 1.734 | 1.474 | 1.094 | 1.0 | 1.327 | 1.206 | 1.076 |
| ***Sepsis/Pneumonia mortality*** | | | | | | | | | | | |
| CALLY | 1.032 | 1.355 | 1.208 | 1.806 | 1.861 | 1.373 | 1.109 | 1 | 1.32 | 1.19 | 1.012 |
| PNI | 1.051 | 1.304 | 1.196 | 1.762 | 1.809 | 1.385 | 1.11 | 1 | 1.22 | 1.234 | 1.012 |
| CONUT | 1.014 | 1.272 | 1.216 | 1.752 | 1.767 | 1.361 | 1.099 | 1 | 1.248 | 1.215 | 1.014 |
| CAR | 1.051 | 1.373 | 1.233 | 1.865 | 1.901 | 1.404 | 1.108 | 1 | 1.318 | 1.158 | 1.012 |
| LA | 1.098 | 1.315 | 1.167 | 1.842 | 1.818 | 1.363 | 1.104 | 1 | 1.261 | 1.247 | 1.011 |

Abbreviation: CVD, cerebrovascular diseases; Dementia, severe dementia; CFS, Clinical Frailty Scale; Asp, aspiration pneumonia; IHD, ischemic heart disease; PEG, percutaneous endoscopic gastrostomy; TPN, total parenteral nutrition; Oral, oral intake recovery; CALLY,C-reactive protein-albumin-lymphocyte index;PNI,prognostic nutritional index;CONUT,controlling nutritional status;CAR,C-reactive protein/albumin ratio;LA,Lymphocyte-to-Albumin.

**Table S3. Nutritional indicators and risk of all-cause mortality**

| **Variable** | **n.total** | **n.event(%)** | **All-cause mortality** | | | |
| --- | --- | --- | --- | --- | --- | --- |
|  |  |  | **Model1** | | **Model2** | |
|  |  |  | **HR (95%CI)** | ***P*-value** | **HR (95%CI)** | ***P*-value** |
| ***lnCALLY*** |  |  |  |  |  |  |
| lnCALLY | 236 | 131 (55.5) | 0.75 (0.68~0.83) | <0.001 | 0.8 (0.72~0.89) | **<0.001** |
| T1(<0.39) | 79 | 58 (73.4) | 1(Ref) |  | 1(Ref) |  |
| T2(0.39~2.27) | 78 | 48 (61.5) | 0.58 (0.39~0.85) | 0.005 | 0.71 (0.47~1.08) | 0.107 |
| T3(≥2.27) | 79 | 25 (31.6) | 0.24 (0.15~0.38) | <0.001 | 0.36 (0.22~0.6) | <0.001 |
| *P* for Trend |  |  |  | <0.001 |  | **<0.001** |
| ***PNI*** |  |  |  |  |  |  |
| PNI* | 236 | 131 (55.5) | 0.51 (0.43~0.62) | <0.001 | 0.57 (0.47~0.7) | **<0.001** |
| T1(<34.55) | 79 | 63 (79.7) | 1(Ref) |  | 1(Ref) |  |
| T2(34.55~40.78) | 78 | 38 (48.7) | 0.39 (0.26~0.58) | <0.001 | 0.5 (0.33~0.76) | 0.001 |
| T3(≥40.78) | 79 | 30 (38) | 0.26 (0.17~0.41) | <0.001 | 0.43 (0.27~0.69) | <0.001 |
| *P* for Trend |  |  |  | <0.001 |  | **<0.001** |
| ***CONUT*** |  |  |  |  |  |  |
| CONUT | 236 | 131 (55.5) | 1.27 (1.19~1.36) | <0.001 | 1.23 (1.14~1.32) | **<0.001** |
| T1(<4) | 79 | 27 (34.2) | 1(Ref) |  | 1(Ref) |  |
| T2(4~6) | 62 | 34 (54.8) | 1.95 (1.18~3.24) | 0.01 | 1.18 (0.7~1.99) | 0.53 |
| T3(≥6) | 95 | 70 (73.7) | 3.74 (2.39~5.87) | <0.001 | 2.35 (1.45~3.8) | 0.001 |
| *P* for Trend |  |  |  | <0.001 |  | **<0.001** |
| ***CAR*** |  |  |  |  |  |  |
| CAR | 236 | 131 (55.5) | 1.31 (1.16~1.49) | <0.001 | 1.24 (1.07~1.44) | **0.004** |
| T1(<0.16) | 79 | 29 (36.7) | 1(Ref) |  | 1(Ref) |  |
| T2(0.16~0.83) | 78 | 47 (60.3) | 1.92 (1.21~3.05) | 0.006 | 1.66 (1.01~2.71) | 0.044 |
| T3(≥0.83) | 79 | 55 (69.6) | 3.02 (1.92~4.75) | <0.001 | 2.14 (1.34~3.43) | 0.001 |
| *P* for Trend |  |  |  | <0.001 |  | **0.001** |
| ***LA*** |  |  |  |  |  |  |
| LA* | 236 | 131 (55.5) | 0.52 (0.4~0.66) | <0.001 | 0.52 (0.4~0.67) | **<0.001** |
| T1(<28.04) | 79 | 64 (81) | 1(Ref) |  | 1(Ref) |  |
| T2(28.04~46.97) | 78 | 40 (51.3) | 0.36 (0.24~0.54) | <0.001 | 0.37 (0.24~0.56) | <0.001 |
| T3(≥46.97) | 79 | 27 (34.2) | 0.21 (0.14~0.34) | <0.001 | 0.21 (0.13~0.35) | <0.001 |
| *P* for Trend |  |  |  | <0.001 |  | **<0.001** |

Model1: Crude Model, Model2: adjusted for sex,age,CI,dement,IHD,Asp,PEG,TPN,oral intake,CFS. PNI*, PNI per SD;LA*, LA per SD. **Abbreviation:**lnCALLY, ln-transformed C-reactive

**Table S4.Nutritional indicators and risk of sepsis/pneumonia mortality**

| **Variable** | **n.total** | **n.event(%)** | **Sepsis or pneumonia mortality** | | | |
| --- | --- | --- | --- | --- | --- | --- |
|  |  |  | **Model1** | | **Model2** | |
|  |  |  | **HR (95%CI)** | ***P*-value** | **HR (95%CI)** | ***P*-value** |
| ***lnCALLY*** |  |  |  |  |  |  |
| lnCALLY | 236 | 62 (26.3) | 0.76 (0.66~0.88) | <0.001 | 0.83 (0.72~0.97) | **0.015** |
| T1(<0.39) | 79 | 28 (35.4) | 1(Ref) |  | 1(Ref) |  |
| T2(0.39~2.27) | 78 | 21 (26.9) | 0.52 (0.3~0.92) | 0.025 | 0.79 (0.43~1.44) | 0.447 |
| T3(≥2.27) | 79 | 13 (16.5) | 0.26 (0.13~0.5) | <0.001 | 0.42 (0.21~0.83) | 0.013 |
| *P* for Trend |  |  |  | <0.001 |  | **0.014** |
| ***PNI*** |  |  |  |  |  |  |
| PNI* | 236 | 62 (26.3) | 0.48 (0.37~0.63) | <0.001 | 0.54 (0.4~0.73) | **<0.001** |
| T1(<34.55) | 79 | 30 (38) | 1(Ref) |  | 1(Ref) |  |
| T2(34.55~40.78) | 78 | 17 (21.8) | 0.36 (0.2~0.65) | 0.001 | 0.45 (0.24~0.84) | 0.012 |
| T3(≥40.78) | 79 | 15 (19) | 0.27 (0.15~0.51) | <0.001 | 0.5 (0.25~0.99) | 0.047 |
| *P* for Trend |  |  |  | <0.001 |  | **0.026** |
| ***CONUT*** |  |  |  |  |  |  |
| CONUT | 236 | 62 (26.3) | 1.3 (1.17~1.43) | <0.001 | 1.25 (1.12~1.4) | **<0.001** |
| T1(<4) | 79 | 13 (16.5) | 1(Ref) |  | 1(Ref) |  |
| T2(4~6) | 62 | 16 (25.8) | 1.91 (0.92~3.99) | 0.084 | 1.2 (0.57~2.55) | 0.634 |
| T3(≥6) | 95 | 33 (34.7) | 3.69 (1.93~7.07) | <0.001 | 2.16 (1.07~4.36) | 0.032 |
| *P* for Trend |  |  |  | <0.001 |  | **0.022** |
| ***CAR*** |  |  |  |  |  |  |
| CAR | 236 | 62 (26.3) | 1.29 (1.07~1.56) | 0.007 | 1.17 (0.94~1.46) | 0.164 |
| T1(<0.16) | 79 | 14 (17.7) | 1(Ref) |  | 1(Ref) |  |
| T2(0.16~0.83) | 78 | 23 (29.5) | 1.95 (1~3.79) | 0.05 | 1.99 (0.98~4.04) | 0.059 |
| T3(≥0.83) | 79 | 25 (31.6) | 2.87 (1.48~5.53) | 0.002 | 1.96 (0.99~3.88) | 0.055 |
| *P* for Trend |  |  |  | 0.001 |  | 0.068 |
| ***LA*** |  |  |  |  |  |  |
| LA* | 236 | 62 (26.3) | 0.47 (0.33~0.68) | <0.001 | 0.49 (0.33~0.73) | **<0.001** |
| T1(<28.04) | 79 | 29 (36.7) | 1(Ref) |  | 1(Ref) |  |
| T2(28.04~46.97) | 78 | 21 (26.9) | 0.42 (0.24~0.74) | 0.003 | 0.55 (0.3~1.01) | 0.053 |
| T3(≥46.97) | 79 | 12 (15.2) | 0.21 (0.11~0.41) | <0.001 | 0.24 (0.11~0.49) | <0.001 |
| *P* for Trend |  |  |  | <0.001 |  | **<0.001** |

Model1: Crude Model, Model2: adjusted for sex,age,CI,dement,IHD,Asp,PEG,TPN,oral intake,CFS. PNI*, PNI per SD;LA*, LA per SD. **Abbreviation:**lnCALLY, ln-transformed C-reactive

**Table S5. Causal mediation analysis of the associations between exposures and mortality outcomes.**

| **Variable** | **Controlled Direct Effect** | | **Pure Natural indirect Effect** | | **Total Effect** | | **Proportion Mediated** | |
| --- | --- | --- | --- | --- | --- | --- | --- | --- |
|  | **HR (95%CI)** | ***P*** | **HR (95%CI)** | ***P*** | **HR (95%CI)** | ***P*** | **HR (95%CI)** | ***P*** |
| ***All-cause mortality*** | | | | | | | | |
| lnCALLY | 0.8305(0.7308~0.9494) | 0.008 | 0.956(0.8951~0.9893) | <0.001 | 0.794(0.6901~0.8833) | <0.001 | 0.1775(0.0373~0.6088) | **<0.001** |
| PNI | 0.9374(0.9072~0.9652) | <0.001 | 0.9895(0.969~1.0017) | 0.108 | 0.9276(0.8982~0.9511) | <0.001 | 0.1358(-0.0213~0.4017) | 0.108 |
| CONUT | 1.1918(1.0872~1.315) | <0.001 | 1.0292(0.9835~1.1006) | 0.152 | 1.2267(1.1491~1.3626) | <0.001 | 0.1538(-0.088~0.4935) | 0.152 |
| CAR | 1.1873(0.9778~1.4198) | 0.076 | 1.0706(1.0236~1.1777) | <0.001 | 1.2712(1.0898~1.5108) | 0.016 | 0.3093(0.0726~1.0547) | **0.016** |
| LA | 0.9769(0.9618~0.9879) | <0.001 | 0.9974(0.993~0.9997) | 0.032 | 0.9744(0.9586~0.9849) | <0.001 | 0.0974(0.0109~0.2825) | **0.032** |
| ***Sepsis or pneumonia mortality*** | | | | | | | | |
| lnCALLY | 0.8635(0.7228~1.044) | 0.132 | 0.9465(0.8551~0.9937) | 0.044 | 0.8173(0.6861~0.9568) | 0.012 | 0.2529(-0.0128~1.6557) | 0.056 |
| PNI | 0.9305(0.8863~0.9845) | 0.008 | 0.9894(0.9587~1.0079) | 0.2 | 0.9206(0.8778~0.9676) | 0.004 | 0.1247(-0.099~0.6798) | 0.204 |
| CONUT | 1.2128(1.0127~1.421) | 0.036 | 1.0333(0.9748~1.1484) | 0.216 | 1.2532(1.0865~1.4589) | <0.001 | 0.1595(-0.1348~0.8958) | 0.216 |
| LA | 0.9756(0.9523~0.9928) | 0.004 | 0.997(0.9897~1.0005) | 0.076 | 0.9727(0.9499~0.9876) | <0.001 | 0.1061(-0.016~0.4998) | 0.076 |

Abbreviations: lnCALLY = natural log–transformed CALLY (see Methods for exact variable definition); PNI = prognostic nutritional index; CONUT = controlling nutritional status score; CAR = C-reactive protein/albumin ratio; LA = lymphocyte/albumin ratio; HR = hazard ratio; CI = confidence interval. Notes: Models and mediation analyses were adjusted for the covariates listed in the Methods section. Estimates are shown for two outcomes: all-cause mortality and sepsis or pneumonia mortality. Adjusted for sex,age,CI,dement,IHD,Asp,PEG,TPN,oral intake,CFS.

**Table S6. ROC curves of five nutritional indexes and all-cause mortality in older patients with dysphagia.**

| **Model** | **AUC(95%CI)** | **Specificity** | **Sensitivity** | **NPV** | **PPV** | **Youden Index** | **NRI** | | **IDI** | |
| --- | --- | --- | --- | --- | --- | --- | --- | --- | --- | --- |
|  |  |  |  |  |  |  | **95%CI** | ***P*** | **95%CI** | ***P*** |
| Alb | 0.7029  (0.636 ~ 0.7698) | 0.66 | 0.69 | 0.63 | 0.71 | 0.34 | Ref |  | Ref |  |
| CALLY | 0.6931  (0.6248~ 0.7614) | 0.52 | 0.81 | 0.69 | 0.68 | 0.33 | 0.031（0.002~0.078） | 0.024 | 0.162（-0.005~0.293） | 0.06 |
| PNI | 0.7211  (0.6561~ 0.7862) | 0.65 | 0.69 | 0.63 | 0.71 | 0.34 | 0.178（0.001~0.326） | **0.048** | 0.04（0.0~0.085） | **0.048** |
| CONUT | 0.7117  (0.6468~ 0.7766) | 0.76 | 0.53 | 0.57 | 0.74 | 0.3 | 0.236（0.084~0.366） | **0.004** | 0.046（0.009~0.096） | **0.008** |
| CAR | 0.6644  (0.5940~ 0.7348) | 0.68 | 0.63 | 0.59 | 0.71 | 0.3 | 0.013（-0.117~0.165） | 0.615 | 0.001（-0.003~0.021） | 0.391 |
| LA | 0.7082  (0.6421~ 0.7742) | 0.82 | 0.53 | 0.59 | 0.79 | 0.35 | 0.208（0.008~0.348） | **0.032** | 0.044（0.003~0.093） | **0.04** |

**Table S7. ROC curves of five nutritional indexes and sepsis or pneumonia mortality in older patients with dysphagia.**

| **Model** | **AUC(95%CI)** | **Specificity** | **Sensitivity** | **NPV** | **PPV** | **Youden Index** | **NRI** | | **IDI** | |
| --- | --- | --- | --- | --- | --- | --- | --- | --- | --- | --- |
|  |  |  |  |  |  |  | **95%CI** | ***P*** | **95%CI** | ***P*** |
| Alb | 0.6399  (0.5607 ~ 0.7191) | 0.54 | 0.74 | 0.85 | 0.37 | 0.28 | Ref |  | Ref |  |
| CALLY | 0.5992  (0.5193 ~ 0.6791) | 0.44 | 0.74 | 0.83 | 0.32 | 0.18 | 0.146  (-0.227~0.279) | 0.251 | 0.006  (-0.01~0.021) | 0.196 |
| PNI | 0.6433  (0.5651 ~ 0.7216) | 0.66 | 0.58 | 0.81 | 0.38 | 0.24 | 0.191  (-0.026~0.372) | 0.064 | 0.031  (0.001~0.084) | 0.048 |
| CONUT | 0.6398  (0.5578 ~ 0.7219) | 0.74 | 0.47 | 0.8 | 0.39 | 0.21 | 0.228  (-0.03~0.421) | 0.088 | 0.033  (0.002~0.111) | 0.032 |
| CAR | 0.5857  (0.5045 ~ 0.6668) | 0.61 | 0.55 | 0.79 | 0.34 | 0.16 | -0.065  (-0.193~0.265) | 1.19 | -0.001  (-0.004~0.029) | 1.377 |
| LA | 0.6202  (0.5407 ~ 0.6997) | 0.39 | 0.81 | 0.85 | 0.32 | 0.19 | 0.197  (-0.06~0.375) | 0.076 | 0.025  (-0.001~0.077) | 0.068 |


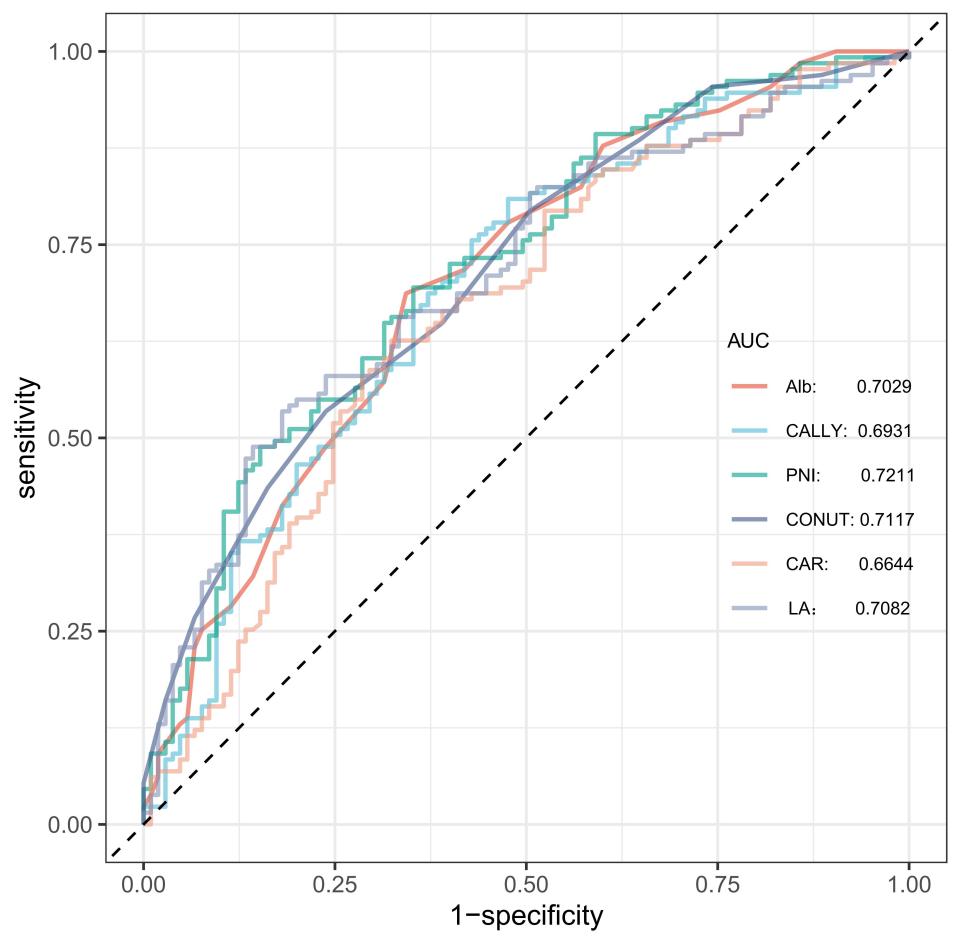

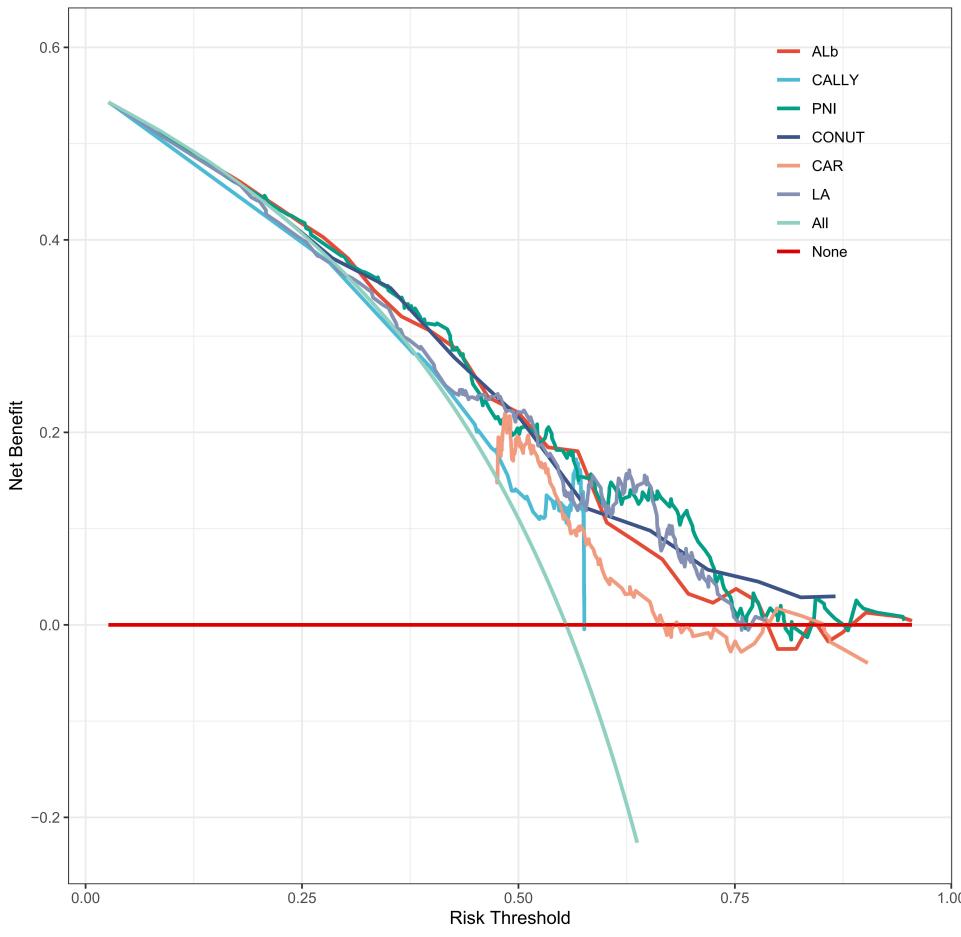


**Figure S1a. ROC analysis for nutritional indicators in patients with all-cause mortality**

**Figure S1b. Calibration curve analysis for the survival prediction of patients with dysphagia in five nutritional indicators.**


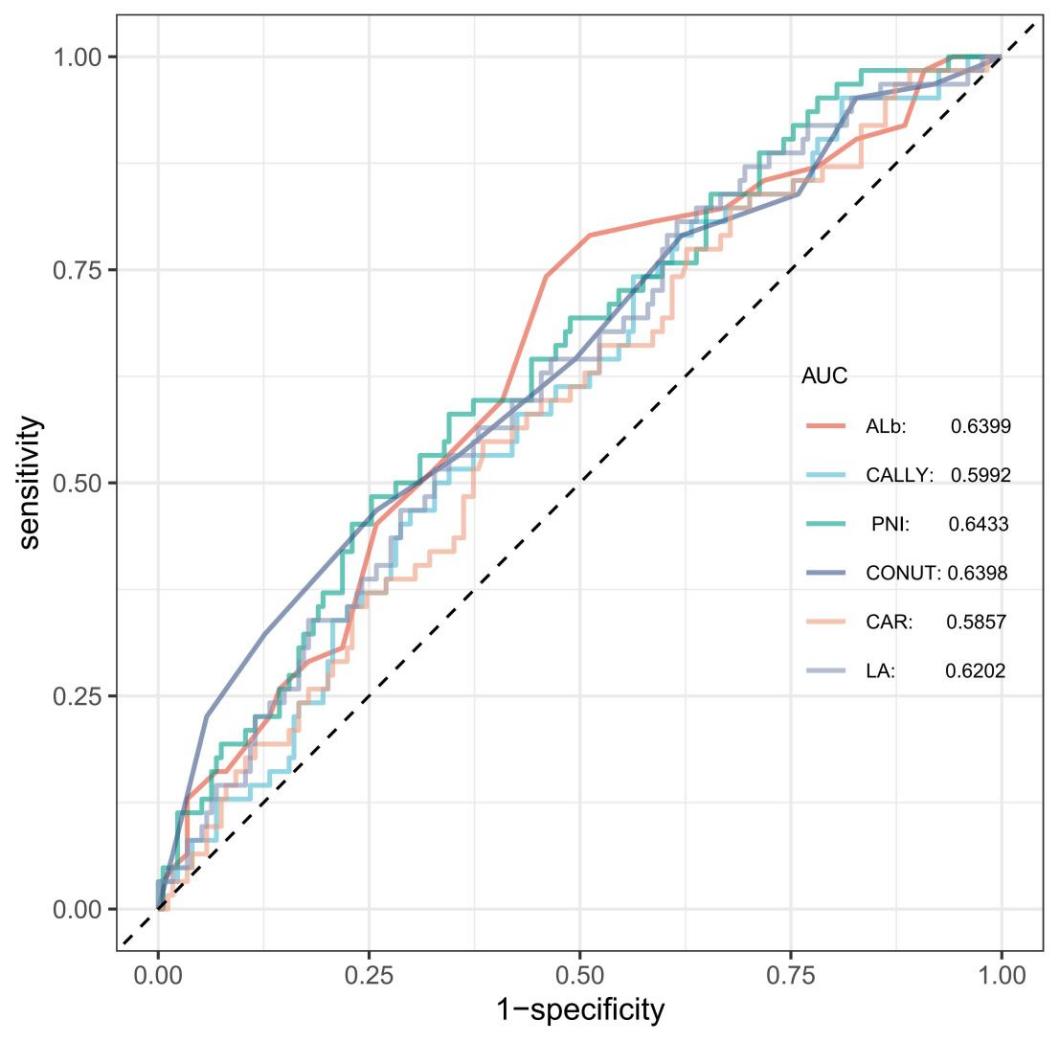

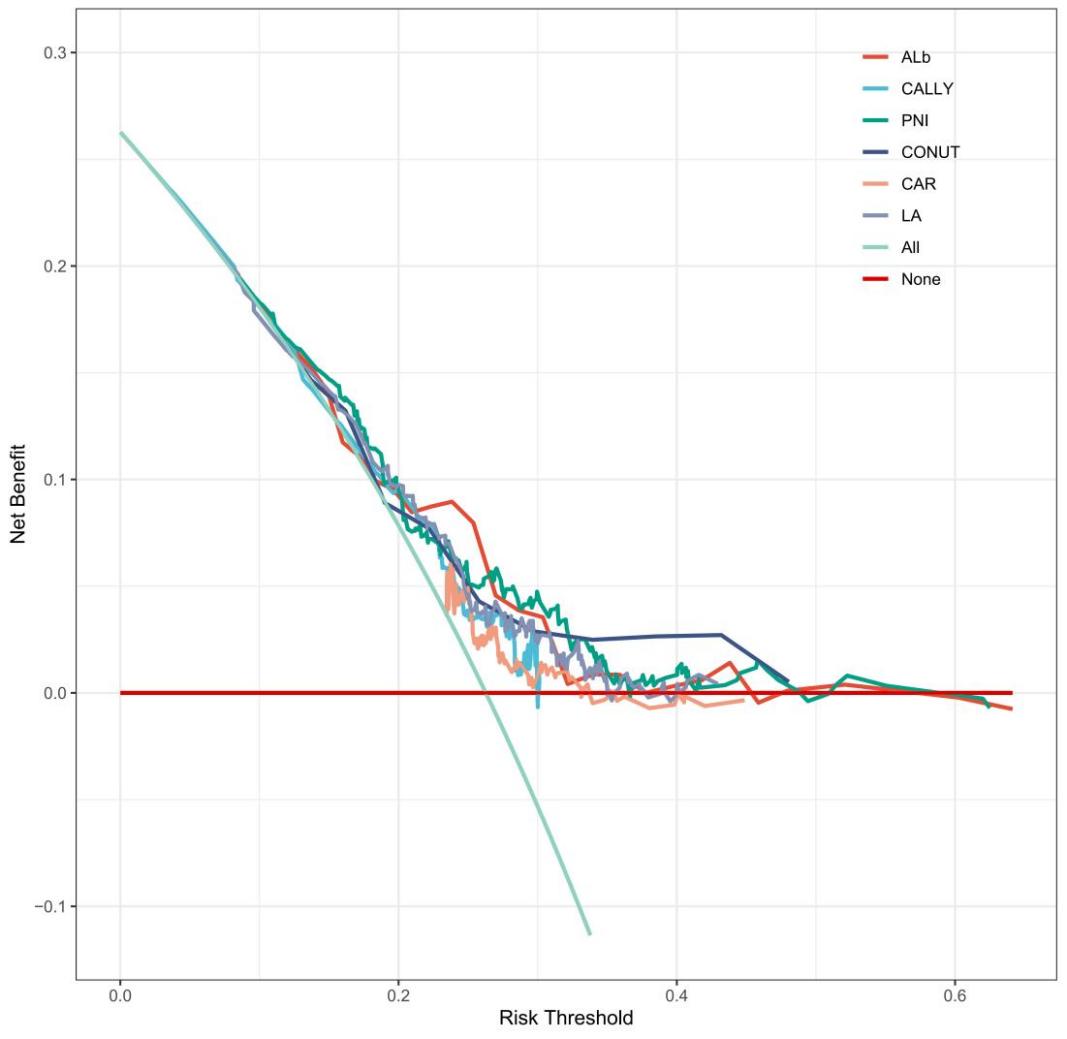


**Figure S2a. ROC analysis for nutritional indicators in patients with sepsis or pneumonia mortality**

**Figure S2b. Calibration curve analysis for the survival prediction of patients with dysphagia in five nutritional indicators.**

| **Variable** | **All-cause mortality** | | | | **Sepsis or pneumonia mortality** | | | |
| --- | --- | --- | --- | --- | --- | --- | --- | --- |
|  | **Model1** | | **Model2** | | **Model1** | | **Model2** | |
|  | **HR (95%CI)** | ***P*-value** | **HR (95%CI)** | ***P*-value** | **HR (95%CI)** | ***P*-value** | **HR (95%CI)** | ***P*-value** |
| ***lnCALLY*** |  |  |  |  |  |  |  |  |
| lnCALLY | 0.74 (0.68~0.82) | <0.001 | 0.79 (0.72~0.88) | **<0.001** | 0.75 (0.66~0.86) | <0.001 | 0.82 (0.71~0.96) | **0.01** |
| T1(<0.45) | 1(Ref) |  | 1(Ref) |  | 1(Ref) |  | 1(Ref) |  |
| T2(0.45~2.31) | 0.53 (0.36~0.77) | 0.001 | 0.63 (0.42~0.94) | 0.023 | 0.51 (0.29~0.89) | 0.017 | 0.72 (0.41~1.29) | 0.27 |
| T3(≥2.31) | 0.23 (0.14~0.37) | <0.001 | 0.35 (0.22~0.57) | <0.001 | 0.25 (0.13~0.49) | <0.001 | 0.42 (0.21~0.83) | 0.012 |
| *P* for Trend |  | <0.001 |  | **<0.001** |  | <0.001 |  | **0.012** |
| ***PNI*** |  |  |  |  |  |  |  |  |
| PNI* | 0.51 (0.43~0.61) | <0.001 | 0.57 (0.47~0.7) | **<0.001** | 0.48 (0.37~0.62) | <0.001 | 0.54 (0.4~0.73) | **<0.001** |
| T1(<34.70) | 1(Ref) |  | 1(Ref) |  | 1(Ref) |  | 1(Ref) |  |
| T2(34.70~41.26) | 0.43 (0.29~0.64) | <0.001 | 0.56 (0.37~0.83) | 0.005 | 0.4 (0.22~0.71) | 0.002 | 0.51 (0.28~0.93) | 0.027 |
| T3(≥40.26) | 0.24 (0.15~0.38) | <0.001 | 0.41 (0.25~0.67) | <0.001 | 0.25 (0.13~0.47) | <0.001 | 0.48 (0.24~0.97) | 0.042 |
| *P* for Trend |  | <0.001 |  | **<0.001** |  | <0.001 |  | **0.023** |
| ***CONUT*** |  |  |  |  |  |  |  |  |
| CONUT | 1.27 (1.19~1.36) | <0.001 | 1.23 (1.14~1.32) | **<0.001** | 1.3 (1.17~1.43) | <0.001 | 1.25 (1.12~1.4) | **<0.001** |
| T1(<4) | 1(Ref) |  | 1(Ref) |  | 1(Ref) |  | 1(Ref) |  |
| T2(4~6) | 1.95 (1.18~3.24) | 0.01 | 1.18 (0.7~1.99) | 0.53 | 1.91 (0.92~3.99) | 0.084 | 1.2 (0.57~2.55) | 0.634 |
| T3(≥6) | 3.74 (2.39~5.87) | <0.001 | 2.35 (1.45~3.8) | 0.001 | 3.69 (1.93~7.07) | <0.001 | 2.16 (1.07~4.36) | 0.032 |
| *P* for Trend |  | <0.001 |  | **<0.001** |  | <0.001 |  | **0.022** |
| ***CAR*** |  |  |  |  |  |  |  |  |
| CAR | 1.28 (1.14~1.44) | <0.001 | 1.28 (1.11~1.48) | **0.001** | 1.26 (1.05~1.5) | 0.011 | 1.19 (0.95~1.47) | 0.125 |
| T1(<0.14) | 1(Ref) |  | 1(Ref) |  | 1(Ref) |  | 1(Ref) |  |
| T2(0.14~0.82) | 2.2 (1.39~3.49) | 0.001 | 2.01 (1.23~3.29) | 0.006 | 2.08 (1.07~4.02) | 0.03 | 1.98 (0.98~4) | 0.058 |
| T3(≥0.82) | 3.35 (2.13~5.27) | <0.001 | 2.63 (1.63~4.24) | <0.001 | 2.97 (1.55~5.72) | 0.001 | 2.09 (1.05~4.16) | 0.036 |
| *P* for Trend |  | <0.001 |  | **<0.001** |  | 0.001 |  | **0.044** |
| ***LA*** |  |  |  |  |  |  |  |  |
| LA* | 0.51 (0.4~0.65) | <0.001 | 0.52 (0.4~0.67) | **<0.001** | 0.48 (0.33~0.68) | <0.001 | 0.5 (0.34~0.73) | **<0.001** |
| T1(<29.95) | 1(Ref) |  | 1(Ref) |  | 1(Ref) |  | 1(Ref) |  |
| T2(29.95~47.42) | 0.41 (0.28~0.6) | <0.001 | 0.38 (0.25~0.58) | <0.001 | 0.47 (0.27~0.81) | 0.007 | 0.55 (0.3~1) | 0.05 |
| T3(≥47.42) | 0.21 (0.13~0.33) | <0.001 | 0.2 (0.12~0.33) | <0.001 | 0.23 (0.12~0.45) | <0.001 | 0.26 (0.13~0.53) | <0.001 |
| *P* for Trend |  | <0.001 |  | **<0.001** |  | <0.001 |  | **<0.001** |

**Table S8. Multivariate Regression Analysis of the Original Data**

**Table S9. Multivariate analysis excluding death within 30 days.**

| **Variable** | **All-cause mortality** | | | | **Sepsis or pneumonia mortality** | | | |
| --- | --- | --- | --- | --- | --- | --- | --- | --- |
|  | **Model1** | | **Model2** | | **Model1** | | **Model2** | |
|  | **HR (95%CI)** | ***P*-value** | **HR (95%CI)** | ***P*-value** | **HR (95%CI)** | ***P*-value** | **HR (95%CI)** | ***P*-value** |
| lnCALLY | 0.78 (0.71~0.86) | <0.001 | 0.82 (0.73~0.92) | **0.001** | 0.77 (0.67~0.9) | 0.001 | 0.83 (0.71~0.98) | **0.023** |
| T1(<0.45) | 1(Ref) |  | 1(Ref) |  | 1(Ref) |  | 1(Ref) |  |
| T2(0.45~2.31) | 0.66 (0.44~1.01) | 0.055 | 0.8 (0.5~1.25) | 0.324 | 0.55 (0.3~1.02) | 0.057 | 0.85 (0.45~1.63) | 0.631 |
| T3(≥2.31) | 0.29 (0.18~0.48) | <0.001 | 0.43 (0.26~0.72) | 0.001 | 0.29 (0.15~0.58) | <0.001 | 0.46 (0.22~0.93) | 0.032 |
| *P* for Trend |  | <0.001 |  | **0.001** |  | <0.001 |  | **0.034** |
| PNI* | 0.55 (0.46~0.67) | <0.001 | 0.6 (0.48~0.75) | **<0.001** | 0.5 (0.38~0.65) | <0.001 | 0.53 (0.38~0.73) | **<0.001** |
| T1(<34.70) | 1(Ref) |  | 1(Ref) |  | 1(Ref) |  | 1(Ref) |  |
| T2(34.70~41.26) | 0.46 (0.3~0.7) | <0.001 | 0.59 (0.38~0.93) | 0.022 | 0.39 (0.21~0.72) | 0.003 | 0.46 (0.24~0.9) | 0.023 |
| T3(≥40.26) | 0.31 (0.2~0.5) | <0.001 | 0.49 (0.3~0.81) | 0.005 | 0.29 (0.15~0.55) | <0.001 | 0.51 (0.25~1.04) | 0.063 |
| *P* for Trend |  | <0.001 |  | **0.004** |  | <0.001 |  | **0.044** |
| CONUT | 1.24 (1.15~1.33) | <0.001 | 1.2 (1.11~1.3) | **<0.001** | 1.28 (1.15~1.42) | <0.001 | 1.24 (1.1~1.4) | **<0.001** |
| T1(<4) | 1(Ref) |  | 1(Ref) |  | 1(Ref) |  | 1(Ref) |  |
| T2(4~6) | 2.03 (1.2~3.44) | 0.008 | 1.23 (0.72~2.12) | 0.447 | 1.98 (0.92~4.25) | 0.079 | 1.27 (0.59~2.78) | 0.541 |
| T3(≥6) | 3.46 (2.15~5.58) | <0.001 | 2.18 (1.31~3.63) | 0.003 | 3.59 (1.81~7.11) | <0.001 | 2.14 (1.02~4.49) | 0.044 |
| *P* for Trend |  | <0.001 |  | **0.002** |  | <0.001 |  | **0.035** |
| CAR | 1.27 (1.1~1.46) | 0.001 | 1.21 (1.02~1.44) | **0.028** | 1.3 (1.07~1.58) | 0.01 | 1.2 (0.94~1.52) | 0.148 |
| T1(<0.14) | 1(Ref) |  | 1(Ref) |  | 1(Ref) |  | 1(Ref) |  |
| T2(0.14~0.82) | 1.92 (1.18~3.11) | 0.008 | 1.67 (1~2.79) | 0.051 | 1.85 (0.92~3.72) | 0.085 | 2 (0.95~4.24) | 0.069 |
| T3(≥0.82) | 2.7 (1.67~4.38) | <0.001 | 1.97 (1.19~3.26) | 0.009 | 2.86 (1.44~5.7) | 0.003 | 2.05 (1~4.22) | 0.052 |
| *P* for Trend |  | <0.001 |  | **0.009** |  | 0.002 |  | 0.06 |
| LA* | 0.59 (0.46~0.76) | <0.001 | 0.56 (0.43~0.73) | **<0.001** | 0.53 (0.36~0.76) | 0.001 | 0.52 (0.35~0.78) | **0.001** |
| T1(<29.95) | 1(Ref) |  | 1(Ref) |  | 1(Ref) |  | 1(Ref) |  |
| T2(29.95~47.42) | 0.43 (0.28~0.66) | <0.001 | 0.41 (0.26~0.65) | <0.001 | 0.48 (0.26~0.87) | 0.016 | 0.62 (0.32~1.19) | 0.15 |
| T3(≥47.42) | 0.26 (0.16~0.42) | <0.001 | 0.23 (0.14~0.39) | <0.001 | 0.24 (0.12~0.49) | <0.001 | 0.26 (0.12~0.57) | 0.001 |
| *P* for Trend |  | <0.001 |  | **<0.001** |  | <0.001 |  | **0.001** |

PNI*, PNI per SD;LA*, LA per SD.

**Table S10. Association between five indices and overall survival and sepsis/pneumonia mortality in patients with dysphagia in different subgroup.**

| **Subgroup** | **n.total** | **n.event_%** | **Model1** | | **Model2** | | **P.for.interaction** |
| --- | --- | --- | --- | --- | --- | --- | --- |
|  |  |  | **HR (95%CI)** | ***P*-value** | **HR (95%CI)** | ***P*-value** |  |
| ***lnCALLY*** | | | | | | | |
| Age(year) |  |  |  |  |  |  | 0.348 |
| Age<90 | 181 | 89 (49.2) | 0.73 (0.65~0.83) | <0.001 | 0.75 (0.66~0.85) | <0.001 |  |
| Age≥90 | 55 | 42 (76.4) | 0.83 (0.71~0.98) | 0.031 | 0.73 (0.57~0.92) | 0.008 |  |
| Sex |  |  |  |  |  |  | 0.214 |
| Male | 95 | 68 (71.6) | 0.7 (0.61~0.82) | <0.001 | 0.71 (0.6~0.83) | <0.001 |  |
| Female | 141 | 63 (44.7) | 0.8 (0.7~0.91) | 0.001 | 0.86 (0.74~1) | 0.05 |  |
| PEG |  |  |  |  |  |  | 0.21 |
| No | 67 | 58 (86.6) | 0.79 (0.65~0.95) | 0.012 | 0.74 (0.61~0.89) | 0.002 |  |
| Yes | 169 | 73 (43.2) | 0.76 (0.67~0.86) | <0.001 | 0.83 (0.72~0.94) | 0.004 |  |
| TPN |  |  |  |  |  |  | **0.021** |
| No | 167 | 94 (56.3) | 0.71 (0.63~0.8) | <0.001 | 0.71 (0.62~0.81) | <0.001 |  |
| Yes | 69 | 37 (53.6) | 0.83 (0.7~0.98) | 0.03 | 0.88 (0.72~1.08) | 0.213 |  |
| ***PNI*** | | | | | | | |
| Age(year) |  |  |  |  |  |  | 0.296 |
| Age<90 | 181 | 89 (49.2) | 0.91 (0.89~0.94) | <0.001 | 0.93 (0.9~0.96) | <0.001 |  |
| Age≥90 | 55 | 42 (76.4) | 0.94 (0.9~0.98) | 0.004 | 0.86 (0.81~0.93) | <0.001 |  |
| Sex |  |  |  |  |  |  | 0.246 |
| Male | 95 | 68 (71.6) | 0.91 (0.87~0.94) | <0.001 | 0.93 (0.9~0.97) | 0.001 |  |
| Female | 141 | 63 (44.7) | 0.92 (0.89~0.95) | <0.001 | 0.92 (0.88~0.95) | <0.001 |  |
| PEG |  |  |  |  |  |  | 0.377 |
| No | 67 | 58 (86.6) | 0.93 (0.89~0.97) | 0.001 | 0.92 (0.88~0.96) | 0.001 |  |
| Yes | 169 | 73 (43.2) | 0.92 (0.89~0.95) | <0.001 | 0.93 (0.9~0.97) | 0.001 |  |
| TPN |  |  |  |  |  |  | 0.688 |
| No | 167 | 94 (56.3) | 0.91 (0.88~0.94) | <0.001 | 0.92 (0.88~0.96) | <0.001 |  |
| Yes | 69 | 37 (53.6) | 0.93 (0.89~0.96) | <0.001 | 0.91 (0.87~0.95) | <0.001 |  |
| ***CONUT*** | | | | | | | |
| Age(year) |  |  |  |  |  |  | 0.536 |
| Age<90 | 181 | 89 (49.2) | 1.28 (1.18~1.39) | <0.001 | 1.23 (1.12~1.35) | <0.001 |  |
| Age≥90 | 55 | 42 (76.4) | 1.21 (1.05~1.38) | 0.006 | 1.46 (1.22~1.74) | <0.001 |  |
| Sex |  |  |  |  |  |  | 0.798 |
| Male | 95 | 68 (71.6) | 1.29 (1.16~1.43) | <0.001 | 1.24 (1.1~1.39) | <0.001 |  |
| Female | 141 | 63 (44.7) | 1.24 (1.13~1.36) | <0.001 | 1.23 (1.1~1.36) | <0.001 |  |
| PEG |  |  |  |  |  |  | 0.333 |
| No | 67 | 58 (86.6) | 1.18 (1.05~1.33) | 0.006 | 1.31 (1.13~1.52) | <0.001 |  |
| Yes | 169 | 73 (43.2) | 1.27 (1.16~1.39) | <0.001 | 1.21 (1.09~1.34) | <0.001 |  |
| TPN |  |  |  |  |  |  | 0.579 |
| No | 167 | 94 (56.3) | 1.32 (1.21~1.43) | <0.001 | 1.27 (1.14~1.41) | <0.001 |  |
| Yes | 69 | 37 (53.6) | 1.2 (1.07~1.34) | 0.002 | 1.24 (1.09~1.41) | 0.001 |  |
| ***CAR*** | | | | | | | |
| Age(year) |  |  |  |  |  |  | 0.21 |
| Age<90 | 181 | 89 (49.2) | 1.33 (1.16~1.54) | <0.001 | 1.37 (1.15~1.63) | <0.001 |  |
| Age≥90 | 55 | 42 (76.4) | 1.2 (0.9~1.61) | 0.222 | 1.37 (0.9~2.08) | 0.144 |  |
| Sex |  |  |  |  |  |  | 0.615 |
| Male | 95 | 68 (71.6) | 1.42 (1.18~1.7) | <0.001 | 1.37 (1.12~1.68) | 0.002 |  |
| Female | 141 | 63 (44.7) | 1.24 (1.03~1.5) | 0.024 | 1.23 (0.94~1.61) | 0.127 |  |
| PEG |  |  |  |  |  |  | 0.568 |
| No | 67 | 58 (86.6) | 1.3 (1.01~1.67) | 0.045 | 1.38 (1.05~1.81) | 0.022 |  |
| Yes | 169 | 73 (43.2) | 1.34 (1.14~1.58) | <0.001 | 1.23 (1.02~1.48) | 0.029 |  |
| TPN |  |  |  |  |  |  | **0.027** |
| No | 167 | 94 (56.3) | 1.35 (1.18~1.55) | <0.001 | 1.36 (1.16~1.59) | <0.001 |  |
| Yes | 69 | 37 (53.6) | 1.17 (0.88~1.56) | 0.283 | 0.97 (0.63~1.49) | 0.889 |  |
| ***LA*** | | | | | | | |
| Age(year) |  |  |  |  |  |  | 0.546 |
| Age<90 | 181 | 89 (49.2) | 0.97 (0.96~0.99) | <0.001 | 0.97 (0.96~0.99) | <0.001 |  |
| Age≥90 | 55 | 42 (76.4) | 0.98 (0.96~1) | 0.021 | 0.96 (0.94~0.98) | <0.001 |  |
| Sex | 95 | 68 (71.6) | 0.97 (0.96~0.99) | <0.001 | 0.97 (0.95~0.98) | <0.001 | 0.804 |
| Male | 141 | 63 (44.7) | 0.98 (0.96~0.99) | 0.001 | 0.98 (0.96~0.99) | 0.001 |  |
| Female |  |  |  |  |  |  | 0.071 |
| PEG | 67 | 58 (86.6) | 0.98 (0.96~0.99) | 0.002 | 0.96 (0.95~0.98) | <0.001 |  |
| No | 169 | 73 (43.2) | 0.97 (0.96~0.99) | <0.001 | 0.98 (0.97~0.99) | 0.003 |  |
| Yes |  |  |  |  |  |  | 0.633 |
| TPN | 167 | 94 (56.3) | 0.97 (0.96~0.98) | <0.001 | 0.97 (0.96~0.98) | <0.001 |  |
| No | 69 | 37 (53.6) | 0.98 (0.96~0.99) | 0.008 | 0.97 (0.95~0.99) | 0.001 |  |
| Yes | 69 | 37 (53.6) | 0.98 (0.96~0.99) | 0.008 | 0.97 (0.95~0.99) | 0.001 |  |
